# Risk factors and oral health‐related quality of life: A case–control comparison between patients after a first‐episode psychosis and people from general population

**DOI:** 10.1111/jpm.12820

**Published:** 2022-02-02

**Authors:** Sonja Kuipers, Stynke Castelein, Hans Barf, Linda Kronenberg, Nynke Boonstra

**Affiliations:** ^1^ NHL Stenden University of Applied Sciences Leeuwarden The Netherlands; ^2^ University of Groningen Groningen The Netherlands; ^3^ Lentis Psychiatric Institute Groningen The Netherlands; ^4^ Dimence Mental Health Care Deventer Deventer The Netherlands; ^5^ KieN Early Intervention Service Leeuwarden The Netherlands

**Keywords:** mental health nurses, oral health, prevention, psychotic disorders, quality of life, risk factors

## Abstract

**What is known on the subject?:**

- Oral health consists of more than having good teeth; it is an important factor in general health and well‐being. Despite its importance, oral health care is still largely overlooked in mental health nursing.
- There is no research available about oral health risk factors and OHRQoL in patients diagnosed with a psychotic disorder with a psychotic disorder (first‐episode).

**What does this paper add to existing knowledge?:**

- This study provides insight into the severity of the problem. It demonstrates the differences in risk factors and OHRQoL between patients diagnosed with a psychotic disorder (first‐episode) and the general population.
- A negative impact on OHRQoL is more prevalent in patients diagnosed with a psychotic disorder (first‐episode) (14.8%) compared to the general population (1.8%).
- Patients diagnosed with a psychotic disorder (first‐episode) have a considerable increase in odds for low OHRQoL compared to the general population, as demonstrated by the odds ratio of 9.45, which supports the importance of preventive oral health interventions in this group.

**What are the implications for practice?:**

- The findings highlight the need for oral health interventions in patients diagnosed with a psychotic disorder (first‐episode). Mental health nurses, as one of the main health professionals supporting the health of patients diagnosed with a mental health disorder, can support oral health (e.g. assess oral health in somatic screening, motivate patients, provide oral health education to increase awareness of risk factors, integration of oral healthcare services) all in order to improve the OHRQoL.

**Abstract:**

**Introduction**

No research is available about the oral health risk factors and oral health‐related quality of life (OHRQoL) in patients diagnosed with a psychotic disorder.

**Aim**

To compare oral health risk factors and OHRQoL in patients diagnosed with a psychotic disorder (first‐episode) to people with no history of psychotic disorder.

**Method**

A case–control comparison (1:2) multivariable linear regression analysis and an estimation of prevalence of impact on OHRQoL.

**Results**

Patients diagnosed with a psychotic disorder (first‐episode) have lower OHRQoL with more associated risk factors. Of the patients diagnosed with a psychotic disorder (first‐episode), 14.8% reported a negative impact on OHRQoL, higher than the prevalence of 1.8% found in people from the general population.

**Discussion**

The high prevalence rate of a negative impact on OHRQoL in patients diagnosed with a psychotic disorder (first‐episode) shows the importance of acting at an early stage to prevent a worse outcome.

**Implications for practice**

The findings highlight the need for oral health interventions in patients diagnosed with a psychotic disorder (first‐episode). Mental health nurses, as one of the main health professionals supporting the health of patients diagnosed with a mental health disorder, can support oral health (e.g. assess oral health in somatic screening) in order to improve the OHRQoL.

## 1 INTRODUCTION

Oral health is an important factor in general health and well‐being (Petersen, 2005). The WHO emphasizes that oral health is essential to general health (Petersen, 2003) and oral health is a determining factor for quality of life (Petersen, 2010). In the last years, oral health is improved in the general population, but vulnerable patients (e.g. patients diagnosed with a mental health disorder) have not benefited of the worldwide improvement in oral health and remain disadvantaged (Kisely et al., 2015). Impacts of diseases are categorized in the WHO’s international classification of general health (WHO, 2001) and are categorized in a hierarchy, ranging from internal symptoms, which primarily affect the individual (e.g. pain), to limitations that are (also) associated with social roles (e.g. family). Poor oral health has a significant impact on the individual and his environment (Petersen, 2010).

Since 1995, the Healthcare Institute of the Netherlands has periodically examined the development of oral health and the preventive dental behaviour of juveniles (Schuller et al., 2018). The outcomes for 17‐ and 23‐year‐olds showed that oral health had stagnated or even deteriorated compared to the same study in 2011. Some differences, in all age categories, are attributable to socioeconomic status (SES): the lower the SES group, the poorer the oral health. Furthermore, the increase in (erosive) dental wear is a cause for concern: 20% of the 17‐year‐olds and more than half of the 23‐year‐olds show wear and tear of the dental bone. Of these groups, 13% of the young adults (>17 years) indicated that they had occasionally postponed dental treatment due to financial considerations. Some young adults will receive information on oral health from their oral health professionals, however, not on a structural basis. Another part of the group may not receive adequate information, which, from the perspective of public oral health, is an alarming development (Schuller et al., 2018).

In the Netherlands, there are guidelines for oral health and oral health care in young children; however, no guidelines are available for oral health in (vulnerable) young adults, e.g. patients diagnosed with a psychotic disorder (first‐episode).

### 1.1 Background oral health in first‐episode psychosis

To date, no research has been conducted on oral health in patients diagnosed with a psychotic disorder (first‐episode). A recent study indicates that patients are hardly concerned with their oral health, there is a lack of awareness and patients are not able to adequately attend to their oral health (Kuipers et al., 2018). Studies on patients diagnosed with a severe mental illness (SMI) showed poor oral healthcare, and highlight the importance of paying attention to oral healthcare (De Hert et al., 2010; Lam et al., 2019). Poor oral health in patients diagnosed with SMI is associated with chronic diseases, e.g. diabetes or cardiovascular diseases (De Hert et al., 2010). Moreover, the prevalence of diabetes in patients diagnosed with schizophrenia is two to three times higher than in the general population (Annamalai & Tek, 2015; De Hert et al., 2010; Mitchell et al., 2013). A meta‐analysis among studies of patients diagnosed with SMI showed a 53% higher risk of having cardiovascular disease (CVD), a 78% higher risk for developing CVD and an 85% higher risk of death from CVD, compared to the regionally matched general population (Correll et al., 2017). Thus, due to the high risks of developing diabetes and/or CVD in patients diagnosed with SMI, it is important to gain insight into oral health‐related risk factors and OHRQoL in patients diagnosed with a psychotic disorder (first‐episode).

### 1.2 1.1.1Risk factors influencing oral health care

Risk factors for poor oral health are related to lifestyle in patients diagnosed with a mental health disorder (e.g. smoking, using alcohol or illicit drugs), side effects (e.g. xerostomia) or (antipsychotic) medication, and consumption of sugary food/drinks (Kisely et al., 2015; Kuipers et al., 2018; McCreadie et al., 2004a; Rossow, 2020).

The Ivory Cross is the Dutch scientific association for the prevention of dental and oral health problems in the Netherlands. They advise brushing two times a day with fluoride toothpaste, for at least two minutes. The use of dental aids (e.g. toothpicks) is also recommended (Ivory Cross, 2011). This advice is substantiated by evidence‐based practice or expert opinions.

Due to poor oral health habits and risk factors related to lifestyle, the influence on patients’ oral health increases the needs for regular check‐ups (e.g. dentist or dental hygienist) (Giannobile et al., 2013; Mitchell et al., 2013; Rossow, 2020). Young adults are advised to pay preventive visits to the dentist and dental hygienist at least once a year (Giannobile et al., 2013).

The accessibility of oral health services and finances (e.g. sufficient money, insurance) have also been determined as risk factors in patients diagnosed with a psychotic disorder (first‐episode) or SMI (Kuipers et al., 2018; Lam et al., 2019; Petersen, 2005). A sample of outpatients diagnosed with SMI show that financial barriers remain a major hurdle to reduce the unmet needs (of dental care) (Lam et al., 2019).

There is no research known that gives insight into the risk factors and oral health‐related quality of life in patients diagnosed with a psychotic disorder (first‐episode) compared to individuals in the general population without any history of a psychotic disorder.

### 1.3 Objectives

This study aims to compare risk factors and oral health‐related quality of life in patients diagnosed with a psychotic disorder (first‐episode) with individuals without any history of psychotic disorder, and to determine risk factors of OHRQoL.

## 2 METHODS

### 2.1 Study design

A case–control comparison was conducted, using survey methodology. To strive for a representative control group, a ratio of 1:2 was opted for. A 1:2 ratio seems the optimal ratio to improve statistical efficiency and to avoid overmatching (Grimes & Schulz, 2005; Hennessy et al., 1999). According to Grimes and Schulz (2005), avoidance of selection bias is important when choosing a control group. Therefore, case and control groups were matched on age (18–25, 25–30 and 31–35), gender (male/female) and educational degree (low, middle or high as defined by the Dutch Central Bureau of Statistics, 2016). In total, 166 individuals were included in the control group.

#### 2.1.1 Recruitment

We carried out a questionnaire (online as well as on paper) among (1) patients diagnosed with a psychotic disorder (first‐episode) and (2) the general population. Data were collected from September 2016 to November 2018.

Patients diagnosed with a psychotic disorder (first‐episode) were recruited from an early intervention service in Leeuwarden (the Netherlands). Patients with a clinical diagnosis of psychotic disorder according to the DSM 5 (American Psychiatric Association, 2013) were included. McGorry et al. (2007) distinguishes different stages in a psychotic disorder, from stage 0 (increased risk of psychotic disorder) to stage 4 (severe, persistent or unremitting illness, as judged by symptoms, neurocognition and disability criteria). All patients in stage 2 having a first episode of psychosis between 18 and 35 years and able to complete the questionnaire were included. Patients with florid psychosis were excluded. Patients who met the inclusion criteria were informed about the study by their mental health nurse and were asked to complete the questionnaire. A total of 130 patients were eligible for the study of which 49 refused to participate. The 81 remaining patients (response rate =62%) agreed to participate and were included in the study.

The recruitment of the control group was in the same period as the case group, based on quota sampling. Case and control groups were matched on gender, age and educational level (van Stralen et al., 2010). Based on these matching criteria, the control group was recruited from the general population in Leeuwarden (the Netherlands) in shopping malls, on the street, at the University of Applied Sciences, and at sporting associations just until the matching criteria were adequately represented. These respondents were recruited by nursing students (bachelor students in the final phase of their study) under the supervision of a research team (S.K. and N.B.). Individuals from the general population were included if they had no mental health problems.

### 2.2 Data collection

The following data were collected: 1. demographical data; 2. risk factors: a) general lifestyle (smoking, alcohol, drugs, antipsychotic medication, sugary food/drinks); b) oral health behaviour (brushing frequency, brushing duration, cleaning tools); c) prevention (dentist visits, dental hygienist visits), accessibility of oral health services, and financial possibilities (sufficient money, insurance); and 3. oral health‐related quality of life (OHIP‐49).

#### 2.2.1 Sociodemographic data

Sociodemographic information included gender, age, educational level and occupational status. The use of antipsychotic medication was registered as a patient characteristic.

#### 2.2.2 Risk factors

Risk factors were assessed based upon the prior month as recall period for risk factors in lifestyle in general, smoking, alcohol, illicit drugs, antipsychotic medication and consumption of sugary food/drinks (Kisely et al., 2011, 2015; Kuipers et al., 2018; McCreadie et al., 2004b; Rossow, 2020). Risk factors were scored dichotomously (present or absent). If participants marked “yes,” this was noted as risk factor. Regarding the frequency of toothbrushing, brushing less than two times a day was scored as a risk factor (Ivory Cross, 2011). Regarding the brushing time, brushing less than two minutes at a time was scored as a risk factor (Ivory Cross, 2011). When no cleaning tools were used, it was also marked as a risk factor (Ivory Cross, 2011). The frequency of dentist and dental hygienist visits were marked as a risk factor if patients visited the dentist and dental hygienist once a year or less (Giannobile et al., 2013; Kuipers et al., 2018; Schuller et al., 2018). Financial risk factors were marked as a risk factor when respondents stated that they did not have enough money to take care of their oral health or if respondents stated that they had no insurance for dental care (Lam et al., 2019).

#### 2.2.3 *Oral health*‐*related quality of life (OHIP*‐*49)*

The Oral Health Impact Profile‐49 (OHIP‐49) (Slade, 1997; Van Der Meulen et al., 2008) was used as a self‐report questionnaire to assess participants’ OHRQoL over the last month. The OHIP consists of 49 items, distributed among seven dimensions: functional limitation (nine items), physical pain (nine items), psychological discomfort (five items), physical disability (nine items), psychological disability (six items), social disability (five items) and handicap (six items) (Slade, 1997). Respondents were asked how frequently they experienced the phenomenon in the last month and responses were recorded using a 5‐point Likert scale, where higher scores indicate worse functioning (0= never, 1= hardly ever, 2=occasionally, 3=fairly often and 4=very often). OHRQoL impairment was determined by the total OHIP‐49 total score, ranging from 0 (no adverse impacts within the last month) to 196 (all 49 impacts are experienced “very often” within the last month). The OHIP‐49 is reported to be valid and reliable (Slade, 1997; Van Der Meulen et al., 2008). The total OHIP‐49 Cronbach's alpha in the current study was 0.91 for cases and 0.90 for controls. Cronbach's alphas for all subscales (case|control) were satisfactory. For functional limitation 0.79|0.74, physical pain 0.82|0.77, psychological discomfort 0.80|0.85, physical disability 0.71|0.74, psychological disability 0.82|0.91, social disability 0.73|0.79 and handicap 0.71|0.83.

The questions of the 14‐item Oral Health Impact Profile (OHIP‐14) (Slade, 1997; Van Der Meulen et al., 2008) which is the shorter version of the OHIP‐49 were used to calculate the estimation of prevalence of impact on OHRQoL in case and control groups. OHIP‐14 scores were ranging from 0 (no adverse impacts within the last month) to 56 (all 14 impacts are experienced “very often” within the last month). The cut‐off scores for the OHIP‐14 were used (Lam et al., 2019; Sanders et al., 2009; Slade et al., 2004), since cut‐off scores for the OHIP‐49 were never studied in previous research. “A negative impact on OHRQoL” indicates that participants reported in line with existing literature: “occurring fairly often” or “often” on one or more of the OHIP‐14 items (Lam et al., 2019; Sanders et al., 2009; Slade et al., 2004). “No impact on OHRQoL” indicates that participants did report in line with existing literature: “Never,” “Hardly Ever” and “Occasionally” on the OHIP‐14 items. The OHIP‐14 has been demonstrated to be reliable in the Netherlands (Van Der Meulen et al., 2008). Internal reliability in our sample was moderate (Cronbach's alpha 0.71|0.77).

### 2.3 Analysis

Descriptive statistics were used to report the demographic information and risk factors of oral health‐related quality of life. Differences in demographics and risk factors between both study groups were analysed using chi‐square tests (χ^2^) and independent t‐tests. Significant group differences were analysed post hoc with Bonferroni correction. Subscale scores of the dimensions of the OHIP‐49 were calculated by summing the responses to subsets of items. The assumption of normality was tested, leading to the conclusion that data were non‐normally distributed. Mann–Whitney U‐tests were conducted to compare dimensions of the OHIP‐49 and the OHIP‐49 total score between the study groups.

To build a model with risk factors as predictors for OHRQoL, a multiple linear regression was conducted. The predictors that were added in the model had never been studied in other studies. Therefore, forced entry was used as a method (Field, 2013). The sum score of the OHRQoL‐49 was used as the dependent variable. Case and control groups were entered in the first stage of the regression. Risk factors were entered at the second stage, to assess the degree to which the model could explain the variance in total OHRQoL. Preliminary analyses were performed to ensure there was no violation of the assumption of normality, linearity, multicollinearity and homoscedasticity (Field, 2013). Chi‐square test of independence (Phi) was performed to examine the strength of the association between binary and dichotomized risk factors (Appendix 1) When associations between variables were <0.60 and the variance inflation factor (VIF) <2, variables were included in the final two models (Field, 2013). There were no associations between risk factors >.60. The assumption of normal distribution was violated, therefore bootstrap was used, and the 95% CI Bias was corrected and accelerated.

To calculate the estimation of prevalence of impact on OHRQoL in case and control groups, the outcomes of the OHIP‐14 items scale were dichotomized, 0= no impact on OHRQoL (score OHIP‐14= 0), 1= negative impact on OHRQoL (score OHIP‐14 ≥1). Next, cross‐tabulation was used on the outcomes of impact on OHRQoL, measured with Fisher's exact test. Odds ratios and confidence intervals (CIs) were calculated. Statistical significance was defined as *p *≤ 0.05 (α‐level=5%), and a 95% CI was chosen. Statistical Package of the Social Sciences (SPSS, version 26) was used for these analyses (IBM, 2019).

#### 2.3.1 Ethical considerations

The research protocol has been approved by The Medical Ethical Committee in Leeuwarden, the Netherlands (decision no. RTPO979a). Standard rules for good clinical practice and ethical principles that have their origin in the Declaration of Helsinki were followed by informing all participants about the study and their rights, and all subjects gave oral consent to participation (The World Medical Association, 2013).

## 3 RESULTS

Table 1 shows the patient characteristics. The mean age of the participants in the case group (N=81) was 25.9 years. The mean age in the control group (N=166) was 25.0 years. There were no significant differences in case and control groups in gender, age, and educational level, which demonstrates a successful matching process.

**TABLE 1:** Characteristics of case and control groups

Characteristics	Case group		Control group		
	*n*	%	*n*	%	*p*
Age, years; mean (SD)	81	25.9 (4.89)	166	25.0 (4.99)	.98
Gender, male	52	64.2	107	64.5	.97
Education					.81
Low	8	9.9	16	9.6	
Middle	50	61.7	100	60.2	
Higher	23	28.4	50	30.1	
Occupational status^b^	81		166		
School	11	13.6	82	49.4	.00^*^
Work	24	29.6	114	68.7	.00^*^
Volunteer work	18	22.2	21	12.7	.06
Day‐care	19	23.5	2	1.2	.00^*^
Nothing	14	17.3	‐	‐	
Other	9	11.1	14	8.4	.49
Medication^a^, ^b^	66	81.5			
Aripiprazole	5	7.6			
Clozapine	9	16.6			
Haloperidol	2	3			
Lithium	3	4.5			
Olanzapine	25	37.9			
Risperidone	15	22.7			
Quetiapine	3	4.5			
Other	23	34.8			
No antipsychotics	10	18.5			

Note

^*^Statistically significant p‐values (*p *< .05).

^a^ Antipsychotics and other common medication that is related to oral health.

^b^ Option to choose more than one.

### 3.1 Comparison of risk factors

Table 2 shows the results of the chi‐square test, conducted to test the differences between case and control groups on risk factors. First, regarding risk factors in general, we found that there was a statistically significant difference between the case and control groups in smoking, *χ^2^* (1) = (20,51), *p* = <.001. The case group demonstrated to smoke more frequently than the control group. Second, regarding risk factors in dental healthcare behaviour, we found that there was a statistically significant difference between the case and control groups in low frequency of brushing, *χ^2^* (1) = (13.45), *p* = <.001. The case group brushed their teeth less often. Last, regarding financial risk factors, we found that there was a statistically significant difference between the case and control groups in finances, *χ^2^* (1) = (33.87), *p* = <.001. The case group reported more frequently a lack of finances to take care of their teeth compared to the control group. No significant differences were found on other risk factors.

**TABLE 2:** Comparison of oral health risk factors in case and control groups

	Case group (*n* = 81)		Control group (*n* = 166)			
Risk factors	n	%	n	%	χ^2^	*p*
Risk factors in general						
Smoking	43	53.1	40	24.1	20.51	.00^*^
Illicit drugs	9	11.1	22	13.3	0.23	.69
Alcohol	52	64.2	127	76.5	4.13	.05
Sugary food/drinks	64	79.0	119	71.7	1.52	.28
Antipsychotics and other common medication that is related to oral health	66	81.5	N/A	N/A	N/A	
Risk factors dental behaviour						
Low frequency brushing	40	49.4	43	25.9	13.45	.00^*^
Short duration brushing	33	40.7	46	27.7	4.25	.04
Few use of dental aid	31	38.3	60	36.1	0.11	.78
Risk factors preventive care						
Low number of dental visits	36	44.4	73	44.0	0.00	1.00
Low number of dental hygienist visits	67	82.7	143	86.1	0.50	.57
Financial risk factors						
Not enough finances	26	32.1	8	4.8	34.13	.00^*^
No insurance oral care	27	33.3	58	34.9	0.06	.89

Note

^*^Statistically significant p‐values (*p *< .05) and corrected for multiple testing using Bonferroni correction (Bonferroni adjustment for alpha=<.004)

### 3.2 Comparison of dimensions and total score of OHRQoL

Table 3 shows the results of the Mann–Whitney U‐tests, conducted to compare the dimensions and total score of OHRQoL between the study groups. A higher score indicates a poorer OHRQoL.

**TABLE 3:** Dimensions and total score of OHRQoL^a^ in case and control groups

Dimension (N items, min‐max score)	Case (*n *= 81)		Control (*n *= 166)			
	Median	Range	Median	Range	Mann Whitney *U*	*p*
Functional limitation (9 items 0–36)	1	9	0	10	5428.5	.00
Physical pain (9 items, 0–36)	1	18	1	14	6418.0	.54
Psychological discomfort (5 items 0–20)	0	15	0	10	4635.5	.00^*^
Physical disability (9 items 0–36)	0	12	0	12	5622.5	.00^*^
Psychological disability (6 items 0–24)	0	10	0	12	6163.0	.05
Social disability (5 items 0–20)	0	2	0	3	6635.5	.63
Handicap (6 items 0–24)	0	9	0	9	6297.5	.09
OHIP total score (0–196)	5	60	1	50	4659.0	.00^*^

Note

^*^Statistically significant p‐values (*p *< .05) are corrected for multiple testing using Bonferroni correction (Bonferroni adjustment for alpha =<.006)

^a^ As measured on the OHIP‐49 scale 0–196. Higher scores mean lower OHRQoL.

Scores in psychological discomfort of the case group (*Mdn* =0, interquartile range [*IQR*] =3) were higher than those of the control group (*Mdn* =0, *IQR* =2). A Mann–Whitney test indicated that this difference was statistically significant, *U* (*N*_case group_ =81, *N*_control group_=166,) =4635.5, *z* = −4.91, *p* < .001. The scores in physical disability of the case group (*Mdn* =0, *IQR* =1) were higher than those of the control group (*Mdn* =0, *IQR* =1). A Mann–Whitney test indicated that this difference was statistically significant, *U* (*N*_case group_ =81, *N*_control group_=166,) =5622.5, *z* = −3.086, *p* = .002. Last, Mann–Whitney tests indicated that scores in the OHIP‐49 total were higher for the case group (*Mdn* =5, *IQR* =6.5) than for the control group (*Mdn* =1, *IQR* =4), *U* (*N*_case group_ =81, *N*_control group_=166,) =4659.0, *z* = −3.91, *p* < 001.

No significant differences were found on the other dimensions.

### 3.3 Risk factors associated with OHRQoL

A multiple regression with forced entry was used to predict risk factors on OHRQoL (Table 4). In the first block, the study group was significantly associated with the value of OHRQoL, *F*(1, 244)= 6.85, *p *< .01, *R* ^2^=.03, *R* ^2^_ajusted_=.02. The case–control group was a significant predictor of OHRQoL, *ß*= −.17, *t*(244)= −2.62 = *p* = .009. The control group corresponded, on average, to a lower score in OHRQoL score of 2.89 points, *B*= −2.89, 95% CI [−5.54,‐.49]. Lower score means better OHRQoL.

**TABLE 4:** Multivariable model of risk factors associated with OHRQoL, with 95% bias corrected and accelerated confidence intervals (CI) (bias corrected and accelerated bootstrap, based on 1000 bootstrap sample; N = 247)

Variable	Model 1			Model 2		
	B	95% CI for B		B	95% CI for B	
		LL	UL		*LL*	*UL*
Constant	6.89	4.93	9.29	3.82	−1.67	9.45
Case–control group^a^	**−2.89** *	−5.54	−.49	.24	−4.15	4.83
Smoking^b^				1.25	−1.32	3.86
Alcohol^b^				**2.34** *	.33	4.51
Illicit drugs^b^				**−3.43** **	−5.58	−1.31
Sugary food/drinks^b^				.98	−.62	2.53
Antipsychotics and other medication related to oral health				2.48	−1.98	7.74
Low frequency brushing^b^				1.59	−.62	3.78
Short duration brushing^b^				1.79	−.53	4.48
Few use of dental aid^b^				−1.50	−3.54	.63
Low dental visits^b^				.85	−1.14	2.85
Low dental hygienist visits^b^				−2.58	−6.76	.83
Not enough finances^b^				.59	−2.64	4.32
No insurance oral health^b^				**−2.67** **	−4.43	−1.04
*R^2^*	.03			.14		
*ΔR^2^*	.02			.09		
*F*	**6.85** *			**2.78** **		

Note

Significant coefficients are displayed in bold. We examined the impact of risk factors on OHRQoL. In model 1, we entered case–control group as predictor. In model 2, we entered the risk factors as predictor.

^a^ Case =0, control =1.

^b^ No risk factor =0, risk factor =1.

* *p* < .05

** *p* < .01.

The multiple linear regression revealed in block 2, introducing the risk factors to the regression model, significantly predicted the value of OHRQoL, *F*(12, 232)= 2.78, *p* = .006, *R* ^2^=.14, *R* ^2^_ajusted_=.09. Drinking alcohol was a significant predictor of OHRQoL, *ß*=.13, *t*(229)=1.82 = *p* = .036. Drinking alcohol as risk factor corresponded, on average, to a higher score in OHRQoL score of 2.48 points, *B*= 2.48, 95%CI [−1.98,‐7.74]. Illicit drug use was a significant predictor of OHRQoL, *ß*= −.14, *t* (232)= −2.08= *p* = .007. Illicit drug use as risk factor corresponded, on average, to a lower score in OHRQoL score of 3.43 points, *B*= −3.43, 95%CI [−5.58, −1.31]. Having an insurance for oral health was a significant predictor of OHRQoL, *ß*= −.15, *t*(232)= −2.44= *p* = .006. Not having an insurance for oral health as risk factor corresponded, on average, to a lower score in OHRQoL score of 2.67 points, *B*= −2.67, 95%CI [−4.44, −1.04]. The other risk factors were found not to be significant in the model.

### 3.4 Prevalence and odds ratio for the impact on OHRQoL

Statistically, a negative impact on OHRQoL was significantly more prevalent in the case group compared to the control group (14,8% versus 1,8% respectively, *p* < 0.0001, Fisher's exact test) (Table 5). Based on the odds ratio, the odds of a negative impact on OHRQoL in the case group was 9.45 (CI 2.59–34.54, *p* < .001) times higher than in the control group.

**TABLE 5:** Prevalence of impact on OHRQoL in case–control group. N = 247

	Negative impact on OHRQoL	No impact on OHRQoL	Total N
Case group	12 (14.8%)	69 (85.2%)	81 (100%)
Control group	3 (1.8%)	163 (98.2%)	166 (100%)

## 4 DISCUSSION

To the best of our knowledge, this is the first study with a case–control comparison design, providing insight into risk factors and the impact on OHRQoL in patients diagnosed with a psychotic disorder (first‐episode) between 18 and 35 years, compared to peers without a history of a psychotic disorder. Our two main findings were, firstly, OHRQoL was significantly lower among patients diagnosed with a psychotic disorder (first‐episode) than in the general population, and, secondly, of the patients diagnosed with a psychotic disorder (first‐episode) 14.8% reported a negative impact on OHQoL, much higher than the prevalence of 1.8% found in people from the general population. This led to a 9.45 times higher risk of impact on OHRQoL in patients diagnosed with a psychotic disorder (first‐episode), compared to their controls. The width of the CI is large. As large CI led to limited confidence in the magnitude of the detected difference, more research would be required.

This study shows that patients diagnosed with a psychotic disorder (first‐episode), in general, have more risk factors (smoking, sugary food/drinks, low frequency of brushing, short duration of brushing, not enough financial means) compared to their peers. This means that oral health awareness training would be beneficial for all young people, especially those diagnosed with a psychotic disorder (first‐episode). At this moment, no oral health interventions are available. However, oral health education, the use of a mechanical toothbrush, reminder systems and brief motivational interviewing sessions in patients diagnosed with SMI or psychotic disorders showed to be effective (Almomani et al., 2006, 2009; Kuo et al., 2020; de Mey et al., 2016). In all samples, oral health knowledge and oral health status (Quigley Hein plaque index) improved significantly. There is no evidence if OHRQoL improved for these populations.

The results in OHRQoL showed that the case group scored significantly poorer in the dimensions psychological discomfort, physical disabilities, psychological disabilities, and in the overall OHIP‐49 score. This could be an effect of antipsychotic medication; however, the objective of this study could not facilitate adding medication as a confounder. The multiple linear regression analysis showed that 14% of the variance in the outcome could be explained by the variables included in the model. Even though there are significant differences between the two study groups in the outcome for OHRQoL, the factors included in the model have limited exploratory value in explaining outcome differences. Additionally, in this research, patients diagnosed with a psychotic disorder (first‐episode) were included. However, these patients might have been more ill than expected. Furthermore, the independent variables included in the analysis did not constitute all factors affecting the OHRQoL. Considering that the mentioned risk factors explained 14% of variance in the outcome of OHRQoL, more insight is needed to identify additional factors affecting OHRQoL. Introducing the risk factors in stage 2 of the regression, illicit drugs contribute to poor OHRQoL. This concurs with recent studies (Rossow, 2020; Teoh et al., 2019). Using illicit drugs causes xerostomia (dry mouth). Xerostomia is an important risk factor for dental caries. Additionally, xerostomia is a debilitating condition in itself causing discomfort and reduced quality of life (Rossow, 2020; Teoh et al., 2019).

The results of this study show some unexpected outcomes. Introducing the risk factors in stage 2 of the regression, drinking alcohol and not having an insurance for oral health care were beneficial risk factor for improving OHRQoL. The literature has shown individuals with alcohol abuse have been found to be at high risk of oral diseases, regardless of the use of alcohol was combined with drugs or not, but the association between alcohol and OHRQoL is questionable since the association is more related to social circumstances and not directly by alcohol consumption (Hede et al., 2019; Teng et al., 2016). In contrast, current study included patients diagnosed with a psychotic disorder (first‐episode) and their peers and did not include the level of alcohol drinking and social circumstances. Furthermore, having no insurance oral health was an unexpected beneficial risk factor for improving OHRQoL. This is in contrast with the findings of Lam et al. (2019) who state that underserved individuals receiving care for SMI in a public mental health service had low OHQoL, driven by unmet dental care needs and xerostomia.

The unexpected outcomes of the multiple regression might be related to differences in the effects of risk factors on OHRQoL between patients diagnosed with a psychotic disorder (first‐episode) and individuals from the general population. This study focussed on main effects. Future studies with an appropriate sample size should also take interactions between group and other risk factors into account.

### 4.1 Study limitations

This study aimed to compare the risk factors and OHRQoL in patients diagnosed with a psychotic disorder (first‐episode) to individuals from the general population. In this study, 81 patients diagnosed with a psychotic disorder (first‐episode) were successfully matched with 166 individuals from the general population without any history of psychotic disorder. Although this sample provides a realistic representation of people living in Friesland, a rural region in the north of the Netherlands, generalizability to a greater population or more urban settings remains to be investigated.

Self‐assessments were used to gain insight into risk factors and OHRQoL, and it could be possible that socially desirable answers were given in the areas of illicit drug or alcohol use. The influence of self‐report in patients diagnosed with a psychotic disorder (first‐episode) or the general population is not known.

In this study, we dichotomized the risk factors. A limitation could be the level of the risk factors (e.g. how much alcohol). These were not taken into account in this study.

We could not use the OHIP‐49 to calculate the prevalence and the odds ratios in this study as previous studies studied no cut‐off points. These were available for the OHIP‐14 in previous studies (Lam et al., 2019; Sanders et al., 2009; Slade et al., 2004). Therefore, the OHIP‐14 was applied to this part of the analysis. It is unknown if this affected the results.

### 4.2 Recommendations

The findings in this study indicate the importance of using educational and behavioural interventions to improve oral health knowledge and motivation in patients diagnosed with a psychotic disorder (first‐episode). Literature showed effective interventions regarding oral health in patients diagnosed with a psychotic disorder (Almomani et al., 2006, 2009; Kuo et al., 2020; de Mey et al., 2016), however the stage a psychotic disorder was in (McGorry et al., 2007) and the effect thereof on OHRQoL were not identified. There is a need for studies exploring what kind of treatment can improve OHRQoL in young adults in general, and patients diagnosed with a psychotic disorder (first‐episode) especially. This means that further research should be continued, and such research should take the stage of the psychotic disorder and the effect thereof on OHRQoL into account.

The results of this study were discussed with an expert by experience, three mental health nurses and professionals from KieN Early Intervention Service Leeuwarden, the Netherlands. The results of the discussion were that mental health nurses state that there is an unintended lack of awareness among mental health nurses regarding the importance of oral health and oral health care. This is based on a lack of knowledge among mental health nurses, as well as a lack of suitable interventions, to be aware of the risk factors and its influence on oral health. Guidelines for lifestyle and patients diagnosed with a psychiatric disorder are lacking. The only guideline developed for people diagnosed with a severe mental illness states that there must be “some attention to oral health.” Because there are no interventions described for this population (or similar populations), many mental health nurses feel to be shy of action. Therefore, an oral healthcare training for mental health nurses is indicated. These results concur with previous research in patients diagnosed with a severe mental illness (Edward et al., 2012; de Mey et al., 2016) showing that not all mental health nurses routinely address oral health interventions in patients.

Mental health nurses, as one of the main health professionals supporting the health of patients diagnosed with a mental health disorder, can support oral health (e.g. assess oral health in somatic screening, motivate patients, provide oral health education to increase awareness of risk factors, integration of oral healthcare services). To be able to pay attention to the oral health of patients, it is important that nurses are aware about the importance of the topic. Mental health nurses can provide more information on their needs, what their barriers are and their attitude on oral health and related issues of physical health care in mental health services.

In order to develop new evidence‐based nursing interventions in oral health care, it is important to involve mental health nurses, as well as experts by experience from the beginning. A design‐oriented approach is an appropriate iterative way of working in co‐creation and suitable for tackling problems in healthcare interventions (Kuipers et al., 2016; Terlouw et al., 2020). The participatory and iterative method of design‐oriented research gives professionals and experts by experience the opportunity to think along from the very start and to give them a decisive voice in appropriate solution directions that really add value.

The results of this study show that there is a need for a prevention and treatment programme for young adults diagnosed with a psychotic disorder. This programme should include an integrated approach between nurses (in mental health care, general health care and community care) and dental professionals. An oral health programme with advice for treatment and prevention of oral health‐related problems, focusing on all young adults (18–35 years), but specially modified to vulnerable young people.

### 4.3 Implications for mental health nursing

This study describes a sample of 81 patients diagnosed with a psychotic disorder (first‐episode) compared with a matched sample of 166 individuals from the general population without a history with psychotic disorder. This study demonstrates the differences in risk factors and oral health‐related quality of life between patients diagnosed with a psychotic disorder (first‐episode) and the general population. A negative impact on OHRQoL is more prevalent in patients diagnosed with a psychotic disorder (first‐episode) (14.8%) compared to the general population (1.8%). The results of this study support the importance of preventive oral health interventions in patients diagnosed with a psychotic disorder (first‐episode). Mental health nurses, as one of the main health professionals supporting the health of patients diagnosed with a mental health disorder, can support oral health (e.g. assess oral health in somatic screening, motivate patients, provide oral health education to increase awareness of risk factors, integration of oral healthcare services), all in order to improve OHRQoL. At this time, existing interventions in patients diagnosed with SMI or psychotic disorders should be modified and tailored to patients’ individual needs.

### 4.4 Relevance statement

This study demonstrates the differences in risk factors and oral health‐related quality of life between patients diagnosed with a psychotic disorder (first‐episode) and the general population. A negative impact on OHRQOL is more prevalent in patients diagnosed with a psychotic disorder (first‐episode) (14.8%) compared to the general population (1.8%), which support the importance of preventive oral health interventions in patients diagnosed with a psychotic disorder (first‐episode). Mental health nurses, as one of the main health professionals supporting the health of patients diagnosed with a mental health disorder, can support oral health (e.g. assess oral health in somatic screening, motivate patients, provide oral health education to increase awareness), in order to improve OHRQoL.

## 5 FUNDING STATEMENT

We appreciate the financial support of the VCVGZ foundation and the NHL Stenden University for Applied Sciences, Leeuwarden, the Netherlands. It is important to note that these funders had no role in study design or collection, analysis and interpretation of data, or the content of this paper.

## 6 ACKNOWLEDGEMENTS

We would like to thank the patients, participants and students who participated in this study for their contribution. We would like to thank Boudien van der Pol RN at KieN Early Intervention Service for the help in supporting the patients, before and after the data collection.

## CONFLICT OF INTEREST STATEMENT

There are no conflicts of interest associated with this research or paper. The authors acknowledge the funding for this project by the VCVGZ foundation and the NHL Stenden, University for Applied Sciences, Leeuwarden, the Netherlands.

## AUTHORSHIP STATEMENT

In keeping with the latest guidelines of the International Committee of Medical Journal Editors. SK contributed to the data collection, statistics and analysis, interpretation of data, drafted the initial paper, provided intellectual content and revised subsequent drafts to final submission. SC wrote and reviewed the manuscript and discussed the results; HB advised on statistics, analysis and reviewed the manuscript; LK wrote and reviewed the manuscript; NB contributed to the conception and design of the research, undertook data analysis and interpretation of data, revised drafts of the manuscript for intellectual content. All authors read and approved the final manuscript, and are all in agreement with the manuscript. The authors listed all and meet the authorship criteria according to the latest guidelines of the International Committee of Medical Journal Editors. All authors are in agreement with the manuscript.

## Data Availability

The data that support the findings of this study are available on request from the corresponding author. The data are not publicly available due to privacy or ethical restrictions.

## APPENDIX 1

*Correlation Matrix (Phi) of dichotomous risk factors*

**Table:**

		**1**	**2**	**3**	**4**	**5**	**6**	**7**	**8**	**9**	**10**	**11**	**12**
1	Smoking	‐											
2	Illicit Drugs	.27*	‐										
3	Alcohol	.09	.07	‐									
4	Sugary food/drinks	.03	−.11	−.05	‐								
5	Medication	.23^**^	.02	−.20^**^	.04	‐							
6	Brush frequency	.08	.14*	.07	−.01	.17*	‐						
7	Brush duration	.12	−.08	.02	−.01	.17*	.01	‐					
8	Use dental aid	.02	.09	.10	−.05	.05	−.01	.05	‐				
9	Dental visits	−.01	.06	−.02	−.01	.04	.15*	.07	.13*	‐			
10	Dental hygienist visits	−.06	−.01	−.03	−.04	−.05	−.09	.07	−.01	.28^**^	‐		
11	Enough money	.11	−.05	.01	.10	.23^**^	.09	.08	−.06	−.07	.04	‐	
12	Insurance for oral health care	−.06	.03	−.01	−.08	.01	−.03	.02	.08	.16*	.07	.11	‐

* Significance ≤.01; ^**^Significance ≤.05

## References

[jpm12820-bib-0002] Almomani, F. , Brown, C. , & Williams, K. B. (2006). The effect of an oral health promotion program for people with psychiatric disabilities. Psychiatric Rehabilitation Journal, 29(4), 10.2975/29.2006.274.281 16689038

[jpm12820-bib-0003] Almomani, F. , Williams, K. , Catley, D. , & Brown, C. (2009). Effects of an oral health promotion program in people with mental illness. Journal of Dental Research, 88(7), 10.1177/0022034509338156 19605879

[jpm12820-bib-0004] American Psychiatric Association. (2013). Diagnostic and statistical manual of mental disorders (DSM‐5). American Psychiatric Pub.

[jpm12820-bib-0005] Annamalai, A. , & Tek, C. (2015). An overview of diabetes management in schizophrenia patients: Office based strategies for primary care practitioners and endocrinologists. International Journal of Endocrinology, 2015, 10.1155/2015/969182 PMC4386295 25878665

[jpm12820-bib-0006] Correll, C. U. , Solmi, M. , Veronese, N. , Bortolato, B. , Rosson, S. , Santonastaso, P. , Thapa‐Chhetri, N. , Fornaro, M. , Gallicchio, D. , Collantoni, E. , Pigato, G. , Favaro, A. , Monaco, F. , Kohler, C. , Vancampfort, D. , Ward, P. B. , Gaughran, F. , Carvalho, A. F. , & Stubbs, B. (2017). Prevalence, incidence and mortality from cardiovascular disease in patients with pooled and specific severe mental illness: a large‐scale meta‐analysis of 3,211,768 patients and 113,383,368 controls. World Psychiatry, 16(2), 163–180. 10.1002/wps.20420 28498599 PMC5428179

[jpm12820-bib-0007] de Hert, M. , Mauri, M. , Shaw, K. , Wetterling, T. , Doble, A. , Giudicelli, A. , & Falissard, B. (2010). The METEOR study of diabetes and other metabolic disorders in patients with schizophrenia treated with antipsychotic drugs. I. Methodology. International Journal of Methods in Psychiatric Research, 19(4), 10.1002/mpr.322 PMC6878539 20683849

[jpm12820-bib-0008] de Mey, L. , Çömlekçi, C. , de Reuver, F. , van Waard, I. , van Gool, R. , Scheerman, J. F. M. , & van Meijel, B. (2016). Oral Hygiene in Patients With Severe Mental Illness: A Pilot Study on the Collaboration Between Oral Hygienists and Mental Health Nurses. Perspectives in Psychiatric Care, 52(3), 194–200. 10.1111/ppc.12117 25902957

[jpm12820-bib-0009] Dutch Central Bureau of Statistics. (2016). The Dutch Standard Classification of Education. [In Dutch: Standaard onderwijsindeling 2016]. Available at: https://www.cbs.nl/nl‐nl/onze‐diensten/methoden/classificaties/onderwijs‐en‐beroepen/standaard‐onderwijsindeling‐‐soi‐‐f

[jpm12820-bib-0010] Edward, K. L. , Felstead, B. , & Mahoney, A. M. (2012). Hospitalized mental health patients and oral health. Journal of Psychiatric and Mental Health Nursing, 19(5), 419–425. 10.1111/j.1365-2850.2011.01794.x 22070464

[jpm12820-bib-0001] Field, A. (2013). Discovering statistics using IBM SPSS statistics. sage.

[jpm12820-bib-0011] Giannobile, W. V. , Braun, T. M. , Caplis, A. K. , Doucette‐Stamm, L. , Duff, G. W. , & Kornman, K. S. (2013). Patient stratification for preventive care in dentistry. Journal of Dental Research, 92(8), 694–701. 10.1177/0022034513492336 23752171 PMC3711568

[jpm12820-bib-0012] Grimes, D. A. , & Schulz, K. F. (2005). Compared to what? Finding controls for case‐control studies. Lancet, 365(9468), 1429–1433. 10.1016/S0140-6736(05)66379-9 15836892

[jpm12820-bib-0013] Hede, B. , Thiesen, H. , & Christensen, L. B. (2019). A program review of a community‐based oral health care program for socially vulnerable and underserved citizens in Denmark. Acta Odontologica Scandinavica, 77(5), 364–370. 10.1080/00016357.2019.1572921 30777469

[jpm12820-bib-0014] Hennessy, S. , Bilker, W. B. , Berlin, J. A. , & Strom, B. L. (1999). Factors influencing the optimal control‐to‐case ratio in matched case‐ control studies. American Journal of Epidemiology, 149(2), 195–197. 10.1093/oxfordjournals.aje.a009786 9921965

[jpm12820-bib-0060] IBM Corp Released (2019). IBM SPSS Statistics for Windows, Version 26.0. IBM Corp.

[jpm12820-bib-1014] Ivory Cross (2011). Practical guide for those who give professional advice on preventive oral care. [In Dutch: Praktische handleiding voor wie professionele adviezen geeft over preventieve mondzorg]. Available at https://ivorenkruis.org/wpȐcontent/uploads/2021/04/IvK_Advies_Cari_spreventie.pdf

[jpm12820-bib-0015] Kisely, S. , Baghaie, H. , Lalloo, R. , Siskind, D. , & Johnson, N. W. (2015). A systematic review and meta‐analysis of the association between poor oral health and severe mental illness. Psychosomatic Medicine, 77(1), 83–92. 10.1097/PSY.0000000000000135 25526527

[jpm12820-bib-0016] Kisely, S. , Quek, L. H. , Pais, J. , Lalloo, R. , Johnson, N. W. , & Lawrence, D. (2011). Advanced dental disease in people with severe mental illness: Systematic review and meta‐analysis. British Journal of Psychiatry, 199(3), 187–193. 10.1192/bjp.bp.110.081695 21881097

[jpm12820-bib-0017] Kuipers, D. A. , Wartena, B. O. , Dijkstra, B. H. , Terlouw, G. , van T Veer, J. T. B. , van Dijk, H. W. , Prins, J. T. , & Pierie, J. P. E. N. (2016). iLift: A health behavior change support system for lifting and transfer techniques to prevent lower‐back injuries in healthcare. International Journal of Medical Informatics, 96, 11–23. 10.1016/j.ijmedinf.2015.12.006 26797571

[jpm12820-bib-0018] Kuipers, S. , Castelein, S. , Malda, A. , Kronenberg, L. , & Boonstra, N. (2018). Oral health experiences and needs among young adults after a first‐episode psychosis : a phenomenological study. Journal of Psychiatric and Mental Health Nursing, 25(8), 10.1111/jpm.12490 29959829

[jpm12820-bib-0019] Kuo, M. W. , Yeh, S. H. , Chang, H. M. , & Teng, P. R. (2020). Effectiveness of oral health promotion program for persons with severe mental illness: a cluster randomized controlled study. BMC Oral Health, 20(1), 10.1186/s12903-020-01280-7 PMC7590455 33109148

[jpm12820-bib-0020] Lam, P. C. , John, D. A. , Galfalvy, H. , Kunzel, C. , & Lewis‐Fernández, R. (2019). Oral health–related quality of life among publicly insured mental health service outpatients with serious mental illness. Psychiatric Services, 70(12), 10.1176/appi.ps.201900111 31522632

[jpm12820-bib-0021] McCreadie, R. G. , Stevens, H. , Henderson, J. , Hall, D. , McCaul, R. , Filik, R. , Young, G. , Sutch, G. , Kanagaratnam, G. , Perrington, S. , McKendrick, J. , Stephenson, D. , & Burns, T. (2004a). The dental health of people with schizophrenia. Acta Psychiatrica Scandinavica, 110(4), 306–310. 10.1111/j.1600-0447.2004.00373.x 15352933

[jpm12820-bib-0022] McCreadie, R. G. , Stevens, H. , Henderson, J. , Hall, D. , McCaul, R. , Filik, R. , Young, G. , Sutch, G. , Kanagaratnam, G. , Perrington, S. , McKendrick, J. , Stephenson, D. , & Burns, T. (2004b). The dental health of people with schizophrenia. Acta Psychiatrica Scandinavica, 110(4), 10.1111/j.1600-0447.2004.00373.x 15352933

[jpm12820-bib-0023] McGorry, P. D. , Purcell, R. , Hickie, I. B. , Yung, A. R. , Pantelis, C. , & Jackson, H. J. (2007). Clinical staging: a heuristic model for psychiatry and youth mental health. The Medical Journal of Australia, 187(7 Suppl), 10.5694/j.1326-5377.2007.tb01335.x 17908024

[jpm12820-bib-0024] Mitchell, A. J. , Vancampfort, D. , Sweers, K. , Van Winkel, R. , Yu, W. , & de Hert, M. (2013). Prevalence of metabolic syndrome and metabolic abnormalities in schizophrenia and related disorders‐a systematic review and meta‐analysis. Schizophrenia Bulletin, 39(2), 306–318. 10.1093/schbul/sbr148 22207632 PMC3576174

[jpm12820-bib-0025] Petersen, P. E. (2003). The World Oral Health Report 2003: Continuous improvement of oral health in the 21st century ‐ The approach of the WHO Global Oral Health Programme. Community Dentistry and Oral Epidemiology, 31(SUPPL, 1). 10.1046/j.2003.com122.x 15015736

[jpm12820-bib-0026] Petersen, P. E. (2005). Priorities for research for oral health in the 21st century ‐ The approach of the WHO Global Oral Health Programme. Community Dental Health, 22(2). 15984131

[jpm12820-bib-0027] Petersen, P. E. (2010). Improvement of global oral health ‐ the leadership role of the World Health Organization. Community Dental Health, 27(4), 194–199. 10.1922/CDH_2760Petersen05 21473352

[jpm12820-bib-0028] Rossow, I. (2020). Illicit drug use and oral health. Addiction, 10.1111/add.15360 33247857

[jpm12820-bib-0029] Sanders, A. E. , Slade, G. D. , Lim, S. , & Reisine, S. T. (2009). Impact of oral disease on quality of life in the US and Australian populations. Community Dentistry and Oral Epidemiology, 37(2), 171–181. 10.1111/j.1600-0528.2008.00457.x 19175659 PMC3760707

[jpm12820-bib-0030] Schuller, A. A. , Vermaire, E. , van Kempen, I. , van Dommelen, P. , & Verrips, E. (2018). Signalement Mondzorg 2018. .

[jpm12820-bib-0031] Slade, G. D. (1997). Concepts of Oral Health (p. 172). Disease and the Quality of Life.

[jpm12820-bib-0032] Slade, G. D. , Foy, S. P. , Shugars, D. A. , Phillips, C. , & White, R. P. (2004). The impact of third molar symptoms, pain, and swelling on oral health‐related quality of life. Journal of Oral and Maxillofacial Surgery, 62(9), 1118–1124. 10.1016/j.joms.2003.11.014 15346364

[jpm12820-bib-0033] Teng, P. R. , Lin, M. J. , & Yeh, L. L. (2016). Utilization of dental care among patients with severe mental illness: A study of a National Health Insurance database. BMC Oral Health, 16(1), 1–7. 10.1186/s12903-016-0280-2 27585979 PMC5009687

[jpm12820-bib-0034] Teoh, L. , Moses, G. , & McCullough, M. J. (2019). Oral manifestations of illicit drug use. Australian Dental Journal, 64(3), 213–222. 10.1111/adj.12709 31309583

[jpm12820-bib-0035] Terlouw, G. , van ‘t Veer, J. T. B. , Prins, J. T. , Kuipers, D. A. , & Pierie, J. E. N. (2020). Design of a Digital Comic Creator (It’s Me) to Facilitate Social Skills Training for Children With Autism Spectrum Disorder: Design Research Approach. JMIR Mental Health, 7(7), 1–18. 10.2196/17260 PMC7382019 32673273

[jpm12820-bib-0036] The World Medical Association (2013). Declaration of Helsinki. Available at https://www.wma.net/policies‐post/wma‐declaration‐of‐helsinki‐ethical‐principles‐for‐medical‐research‐involving‐human‐subjects/

[jpm12820-bib-0038] van Der Meulen, M. J. , John, M. T. , Naeije, M. , & Lobbezoo, F. (2008). The Dutch version of the Oral Health Impact Profile (OHIP‐NL): Translation, reliability and construct validity. BMC Oral Health, 8(1), 1–7. 10.1186/1472-6831-8-11 18405359 PMC2329613

[jpm12820-bib-0039] van Stralen, K. J. , Dekker, F. W. , Zoccali, C. , & Jager, K. J. (2010). Case‐control studies–an efficient observational study design. Nephron. Clinical Practice, 114(1), 1–4. 10.1159/000242442 19797931

[jpm12820-bib-0040] WHO (2001). World Health Organization. World Report on Child Injury Prevention. https://apps.who.int/iris/bitstream/handle/10665/42407/9241545429.pdf 26269872

